# The diversity of endophytic fungi in Tartary buckwheat (*Fagopyrum tataricum*) and its correlation with flavonoids and phenotypic traits

**DOI:** 10.3389/fmicb.2024.1360988

**Published:** 2024-03-14

**Authors:** Meiqi Chen, Ziqi Ding, Min Zhou, Yukun Shang, Chenglei Li, Qingfeng Li, Tongliang Bu, Zizhong Tang, Hui Chen

**Affiliations:** College of Life Sciences, Sichuan Agricultural University, Ya’an, China

**Keywords:** Tartary buckwheat, endophytic fungi, high-throughput sequencing, flavonoids, phenotypic traits

## Abstract

Tartary buckwheat (*Fagopyrum tataricum*) is a significant medicinal crop, with flavonoids serving as a crucial measure of its quality. Presently, the artificial cultivation of Tartary buckwheat yields low results, and the quality varies across different origins. Therefore, it is imperative to identify an effective method to enhance the yield and quality of buckwheat. Endophytic fungi reside within plants and form a mutually beneficial symbiotic relationship, aiding plants in nutrient absorption, promoting host growth, and improving secondary metabolites akin to the host. In this study, high-throughput sequencing technology was employed to assess the diversity of endophytic fungi in Tartary buckwheat. Subsequently, a correlation analysis was performed between fungi and metabolites, revealing potential increases in flavonoid content due to endophytic fungi such as *Bipolaris*, *Hymenula*, and *Colletotrichum*. Additionally, a correlation analysis between fungi and phenotypic traits unveiled the potential influence of endophytic fungi such as *Bipolaris*, *Buckleyzyma*, and *Trichosporon* on the phenotypic traits of Tartary buckwheat. Notably, the endophytic fungi of the *Bipolaris* genus exhibited the potential to elevate the content of Tartary buckwheat metabolites and enhance crop growth. Consequently, this study successfully identified the resources of endophytic fungi in Tartary buckwheat, explored potential functional endophytic fungi, and laid a scientific foundation for future implementation of biological fertilizers in improving the quality and growth of Tartary buckwheat.

## 1 Introduction

Tartary buckwheat [*Fagopyrum tataricum* (L.) Gaertn.] is a traditional cereal crop in China, originating from the southwestern part of the country (Zhou et al., 2023). Its cultivation has expanded across various Asian countries, including China, India, Nepal, and others (Kreft et al., 2023). China ranks first globally in terms of both cultivation area and yields for Tartary buckwheat, with primary cultivation regions located in Sichuan, Guizhou, Yunnan, Xizang, Qinghai, and other areas (Li N. et al., 2022). Notably, historical records in the “Compendium of Materia Medica” highlight the digestive, heat-alleviating, and swelling-reducing properties of Tartary buckwheat (Yan et al., 2021). Scientific research has further revealed the high nutritional value of Tartary buckwheat, including its abundance of carbohydrates, proteins, polyphenols, and minerals (Zou et al., 2021). Moreover, Tartary buckwheat stands out among other food crops due to its rich content of bioactive compounds such as rutin, quercetin, and other flavonoids, which contribute to its noteworthy therapeutic effects in antioxidant, anticancer, blood sugar-lowering, blood pressure-lowering, and lipid-lowering aspects (Zhu, 2016).

Tartary buckwheat is primarily cultivated in arid and semi-arid regions at altitudes ranging from 1,000 to 3,000 meters (Fan et al., 2019). However, challenging cultivation environments often negatively impact seed germination, leading to lower yields and reduced quality of Tartary buckwheat (Weng et al., 2022). Furthermore, the current cultivation area of Tartary buckwheat in China, approximately 705,000 hm^2^, lags significantly behind other traditional cereal crops such as rice and wheat (Chen, 2018). However, with improving living standards, there is a growing emphasis on health and well-being, resulting in an increased demand for whole grains, including Tartary buckwheat. This surge in demand necessitates an emphasis on higher-quality Tartary buckwheat (Qu, 2021). Therefore, in addition to enhancing cultivation techniques and expanding cultivation areas for Tartary buckwheat, there should be a focus on increasing the content of secondary metabolites to enhance its overall quality and align it with the modern demand for health-conscious food choices. Research suggests that the production of plant secondary metabolites is influenced by environmental factors such as altitude, light exposure, and temperature (Večeřová et al., 2021). Furthermore, it is also influenced by inherent plant characteristics, including endophytic fungi and enzymes (Aktar et al., 2022).

Endophytic fungi are non-pathogenic fungal organisms that reside within plant tissues for extended periods, establishing mutualistic relationships with their hosts (Sun et al., 2022). Previous studies indicate that endophytic fungi play a crucial role in nutrient absorption, host immunity enhancement, and overall growth and development promotion (Poudel et al., 2021; Ding et al., 2022). Moreover, specific endophytic fungi can aid plants in resisting pathogens and adversity, enhancing their environmental adaptability and resilience (Waller et al., 2005). Additionally, certain endophytic fungi contribute to the degradation and recycling of organic substances in the environment, thereby promoting soil health and ecosystem stability (Rouydel et al., 2021). Furthermore, extensive research demonstrates that endophytic fungi significantly influence the production of host secondary metabolites. For instance, the diversity and abundance of endophytic fungi in *Gentiana officinalis* exhibit a notable correlation with the content of four medicinal secondary metabolites (Hou et al., 2022). Similarly, four endophytic fungal strains were screened from rice (*Oryza sativa* L.) that effectively promoted rice growth as well as significantly increased the accumulation of phenolic compounds and anthocyanins in rice (Gateta et al., 2023). Notably, the co-fermentation of the endophytic fungus *Ilyonectria cyclaminicola* with the residue of *Epimedii folium* during the fermentation process enhances the content of total flavonoids and flavonols in the fermentation broth (Guo et al., 2023). Under alkaline stress, endophytic fungi promote the production of secondary metabolites such as phosphorus, polyphenols, and alkaloids in *Hordeum bogdanii*, thus enhancing its alkaline tolerance (Han et al., 2022). In other words, endophytic fungi play a crucial role in helping plants endure adversities by stimulating the production of host metabolites. Notably, studies by Harrison et al. demonstrated the influence of endophytic fungi *Alternaria* on plant phenotypes, such as the promotion of leaf area and plant height in *Astragalus lentiginosus* (Harrison et al., 2021). Despite the plethora of research reports on endophytic fungi, the functions of many of these organisms remain unclear.

Traditional methods in microbial research have historically relied on direct cultivation using culture media, which involves the formation of visible microbial colonies followed by purification and molecular characterization (Wei et al., 2021). However, these methods have significant drawbacks, including time-consuming procedures, operational complexity, sample contamination risks, and notable limitations. It is worth noting that cultivable microorganisms only represent a small fraction (approximately one-tenth) of the total microbial community within a sample (Lahlali et al., 2021; Long et al., 2021). Fortunately, with the rapid advancement of bioinformatics technology, DNA sequencing has achieved remarkable progress, particularly through the utilization of high-throughput sequencing. This innovative approach has become the mainstream technology for studying microbial communities due to its advantages, including cost-effectiveness, shorter processing times, and higher data accuracy, overcoming the limitations associated with traditional methods (Jo et al., 2020). In this study, high-throughput sequencing technology was implemented for the first time to examine the diversity of endophytic fungi in various regions and tissues of Tartary buckwheat. Subsequently, correlation analyses were conducted between endophytic fungi and flavonoid secondary metabolites, as well as phenotypic traits. Through this comprehensive analysis of the approach to find a target endophytic fungal strains with the potential to promote the secretion of flavonoids in Tartary buckwheat and with the potential to promote the growth of Tartary buckwheat. This approach establishes a solid theoretical foundation for the potential utilization of microbial fertilizers and provides new insights for further enhancing the yield and quality of Tartary buckwheat.

## 2 Materials and methods

### 2.1 Acquisition of experimental materials and phenotypic assessment

**Figure 1:**
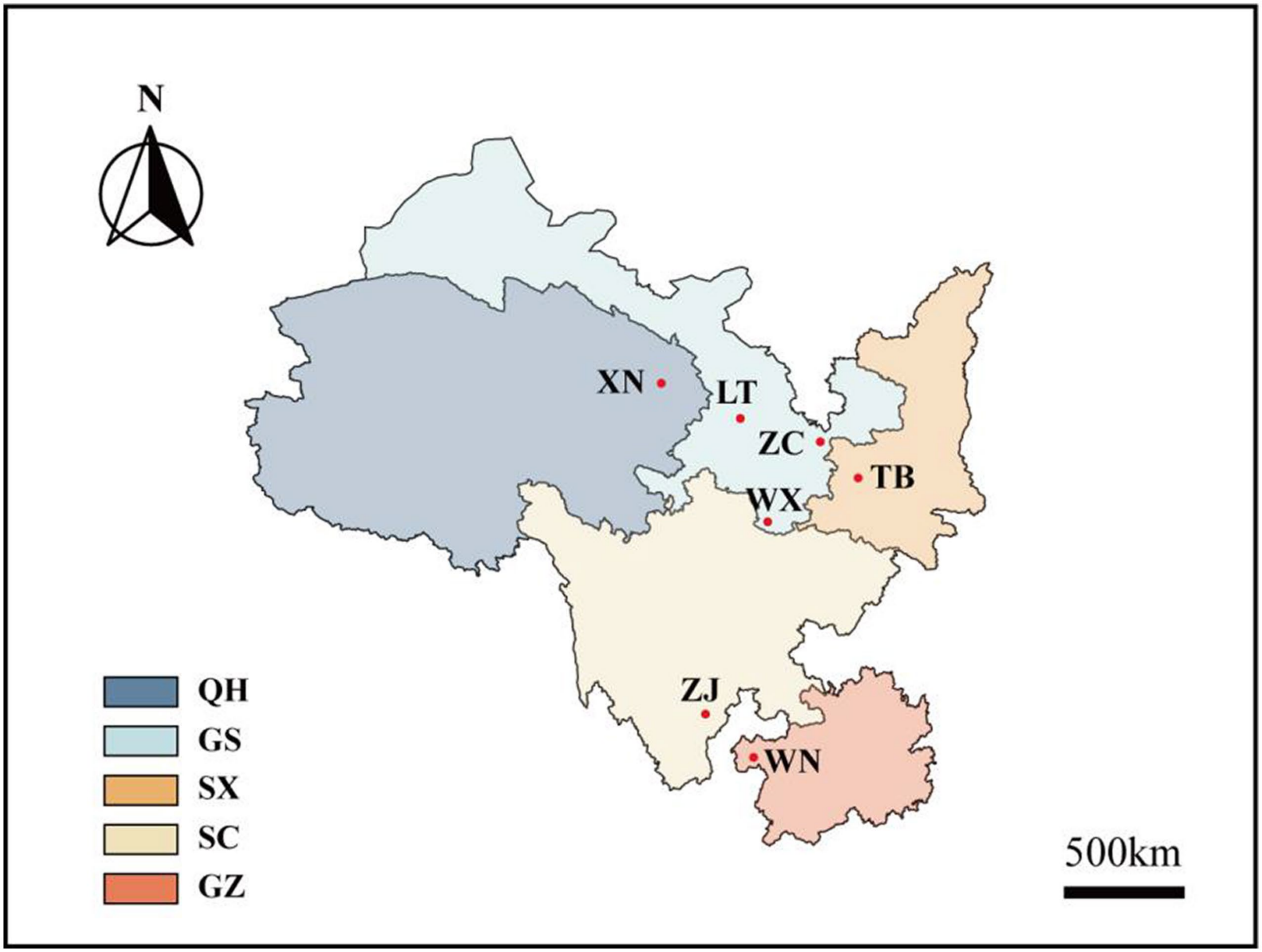
Distribution map of Tartary buckwheat. Xining City (XN), Lintao County (LT), Zhangchuan County (ZC), Wen County (WX), Taibai County (TB), Zhaojue County (ZJ), and Weining County (WN) are the seven wild buckwheat collection sites. Among them, XN belongs to China’s Qinghai Province (QH), LT, ZC, and WX belongs to China’s Gansu Province (GS), TB belongs to China’s Shaanxi Province (SX), ZJ belongs to China’s Sichuan Province (SC), WN belongs to China’s Guizhou Province (GZ).

In June 2022, seven wild *Fagopyrum tataricum* species with distinct phenotypes were collected from the Wild Buckwheat Germplasm Resource Nursery located in Liangshan Yi Autonomous Prefecture, Sichuan Province (longitude: 102°49′E, latitude: 27°59′N, altitude: 2,118 m). These seven materials originate from diverse habitats in five different provinces across China (Figure 1; Supplementary Table S1). Each species consisted of 10 plants, resulting in 70 Tartary buckwheat samples.

A total of eight phenotypic traits were identified in the sample. Plant height and length of middle branches were measured using a tape measure, whereas stem thickness, leaf length, and leaf width were measured using calipers. The branching angle was determined using a protractor. The number of main stem nodes and branches on the main stem were directly observed and counted. Initially, the eight phenotypic traits of Tartary buckwheat samples were determined. Plant height and length of middle branches were measured using a tape measure, while stem thickness, leaf length, and leaf width were measured using calipers. The branching angle was determined using a protractor, and the number of main stem nodes and branches on the main stem were directly observed and counted. Following this, the roots, stems, and leaves of the sample were separated and subjected to surface sterilization treatment within 48 h. This involved soaking them in 75% alcohol for 1 min, followed by immersion in a 2.5% sodium hypochlorite solution for 5–10 min, and rinsing five to six times with sterile water. The Tartary buckwheat roots, stems, and leaves were then divided equally into two parts. One part was stored in sterile self-sealing bags for high-throughput sequencing, while the other part was placed in an oven at a constant temperature of 60°C for drying. Once dried, the material was ground and passed through a 40 mesh sieve to obtain a uniform powder. This powder was used for extracting and quantifying the amount of secondary metabolites in Tartary buckwheat.

### 2.2 Total DNA extraction and high throughput sequencing of Tartary buckwheat

Total genomic DNA samples were extracted utilizing the OMEGA Soil DNA Kit (Omega Bio-Tek, Norcross, GA, United States). Subsequently, the DNA samples were stored at −20°C for further analysis. The quality of the DNA was assessed utilizing a NanoDrop NC2000 spectrophotometer (Thermo Fisher Scientific, Waltham, MA, United States) as well as agarose gel electrophoresis. The fungal internal transcribed spacer (ITS rDNA) was amplified using the primers ITS1F (5′-CTTGGTCATTTAGAGGAAGTAA-3′) and ITS2R (5′-GCTGCGTTCTTCATCGATGC-3′). The resulting PCR amplicons were purified using Vazyme VAHTSTM DNA Clean Beads (Vazyme, Nanjing, China) and quantified utilizing the Quant-iT PicoGreen dsDNA Assay Kit (Invitrogen, Carlsbad, CA, United States). Following individual quantification, the amplicons were pooled in equal amounts and subjected to pair-end 2 × 250 bp sequencing utilizing the Illumina NovaSeq platform with the NovaSeq 6000 SP Reagent Kit (500 cycles) (Shanghai Personal Biotechnology Co., Ltd., Shanghai, China). The raw DNA sequence data were deposited in the National Center for Biotechnology Information Sequence Read Archive database (BioProject accession number: PRJNA1043747).

### 2.3 Determination of total flavonoids and polyphenols in the roots, stems and leaves of Tartary buckwheat

The content of total flavonoids was determined using the sodium nitrite-aluminum nitrate method (Zhao et al., 2016). Tartary buckwheat root and stem leaves weighing 0.3–0.4 g were extracted with 30% ethanol under ultrasonic conditions for 40 min. The resulting supernatant was utilized as the test solution for measurement. To this solution, 0.7 mL of a 5% NaNO_2_ solution was added, followed by the immediate addition of 0.7 mL of a 10% Al(NO_3_)_3_ solution, with subsequent shaking. After 5 min, 5 mL of a 4% NaOH solution was added, mixed, and left to stand for another 5 min. The flavonoid content was determined by measuring the D510 nm value using a UV spectrophotometer and calculating it against a standard curve. The total phenol content was determined using the Folin phenol method (You and Cao, 2013). Specifically, 1 mL of the test solution was mixed with 1 mL of Folin Phenol chromogenic agent and 3 mL of 20% Na_2_CO_3_. The mixture was thoroughly combined and allowed to react in a water bath at 50°C for 30 min. The absorbance was measured at 765 nm, and the total phenol content was calculated against a gallic acid standard curve. All concentration determinations were performed in triplicate, and the average values were taken.

### 2.4 Extraction and content determination of 40 flavonoids from roots, stems, and leaves of ZC and LT

Following the study conducted by Gao et al. (2016), the extraction method was optimized. First, 0.5 g of the crushed dry sample was weighed and mixed with 4 mL of a 1% hydrochloric acid methanol solution. The resulting mixture was vortexed for 1 min and then sonicated in an ice water bath for 30 min. Subsequently, it was centrifuged at 11,000 rpm for 30 min, and the supernatant was carefully removed. This extraction process was repeated once, and the supernatant was combined twice and diluted to a volume of 10 mL. The resulting solution was subjected to quantitative and qualitative analysis after passing through a 0.22-μm filter membrane.

The Agilent 1260 high-performance liquid chromatograph, in series with a 6420A mass spectrometer system, was utilized for the analysis. Various bioactive compounds were separated using a reverse chromatography column, the Agilent ZORBAX Eclipse Plus C18 (3.5 μm, 2.1*150 mm). The chromatographic conditions were based on the method outlined by Aguirre-Hernández et al. (2009). The mobile phase consisted of 0.3% phosphoric acid (A) and acetonitrile (B), with the elution gradient as follows: 0 ∼ 1 min, 80% ~ 10% A; 1 ∼ 5 min, 10% A; 5 ∼ 5.1 min, 10% ~ 80% A; 5.1 ∼ 10 min, 80% A. The flow rate was set at 0.3 mL/min, the injection volume at 3 μL, and the column temperature at 35°C. To establish standard curves of 40 flavonoids, the mobile phase was used to achieve final concentrations of 2 ng/mL, 5 ng/mL, 20 ng/mL, 50 ng/mL, 200 ng/mL, and 500 ng/mL. Following analysis under the aforementioned chromatographic conditions, a standard curve was constructed (Supplementary Figure S1). Qualitative analysis was then conducted by comparing the retention time of the standards with the subsequent detection results. Furthermore, quantitative determination was performed by comparing the concentration and peak area of the reference standard in the chromatography with the constructed standard curve.

The method described by Ma et al. (2022) formed the basis for the detection using mass spectrometry, albeit with slight optimizations. LC–MS analysis was conducted employing a 6420A mass spectrometer (Agilent Technologies, Santa Clara, CA, United States). Details of the selection reaction detection conditions for the protonation and deprotonation of the samples are displayed in Supplementary Table S2. To confirm the presence of flavonoid metabolites, the mass spectra of the total ion chromatography peaks obtained from the extract of Tartary buckwheat roots, stems, and leaves were compared to the mass spectra of standard substances such as rutin, hesperidin, and quercetin.

### 2.5 Bioinformatics and statistical analysis

The raw sequence data were demultiplexed using the demux plugin, followed by primer cutting using the cutadapt plugin (Martin, 2011). Subsequently, the sequences underwent quality filtering, denoising, merging, and chimera removal using the DADA2 plugin (Callahan et al., 2016). The sequence data was analyzed using QIIME2 and R packages (v3.2.0).

To assess ASV-level alpha diversity indices, including the Chao1 richness estimator, Observed species, Shannon diversity index, Simpson index, Pielou’s evenness, and Good’s coverage, the ASV table in QIIME2 was utilized. Beta diversity analysis was conducted using Bray-Curtis metrics to investigate the structural variation of microbial communities among the samples, with the results visualized through principal coordinate analysis (PCoA). The R package “VennDiagram” was employed to generate Venn diagrams, allowing visualization of shared and unique ASVs across samples or groups based on their occurrence, regardless of their relative abundance (Zaura et al., 2009). To detect differentially abundant taxa across groups, Linear Discriminant Analysis Effect Size (LEfSe) was employed, utilizing default parameters (Segata et al., 2011). Additionally, a co-occurrence network was constructed based on the correlation coefficient and visualized using the R packages igraph and ggraph.

## 3 Results

### 3.1 Diversity analysis of endophytic fungi in Tartary buckwheat

**Figure 2:**
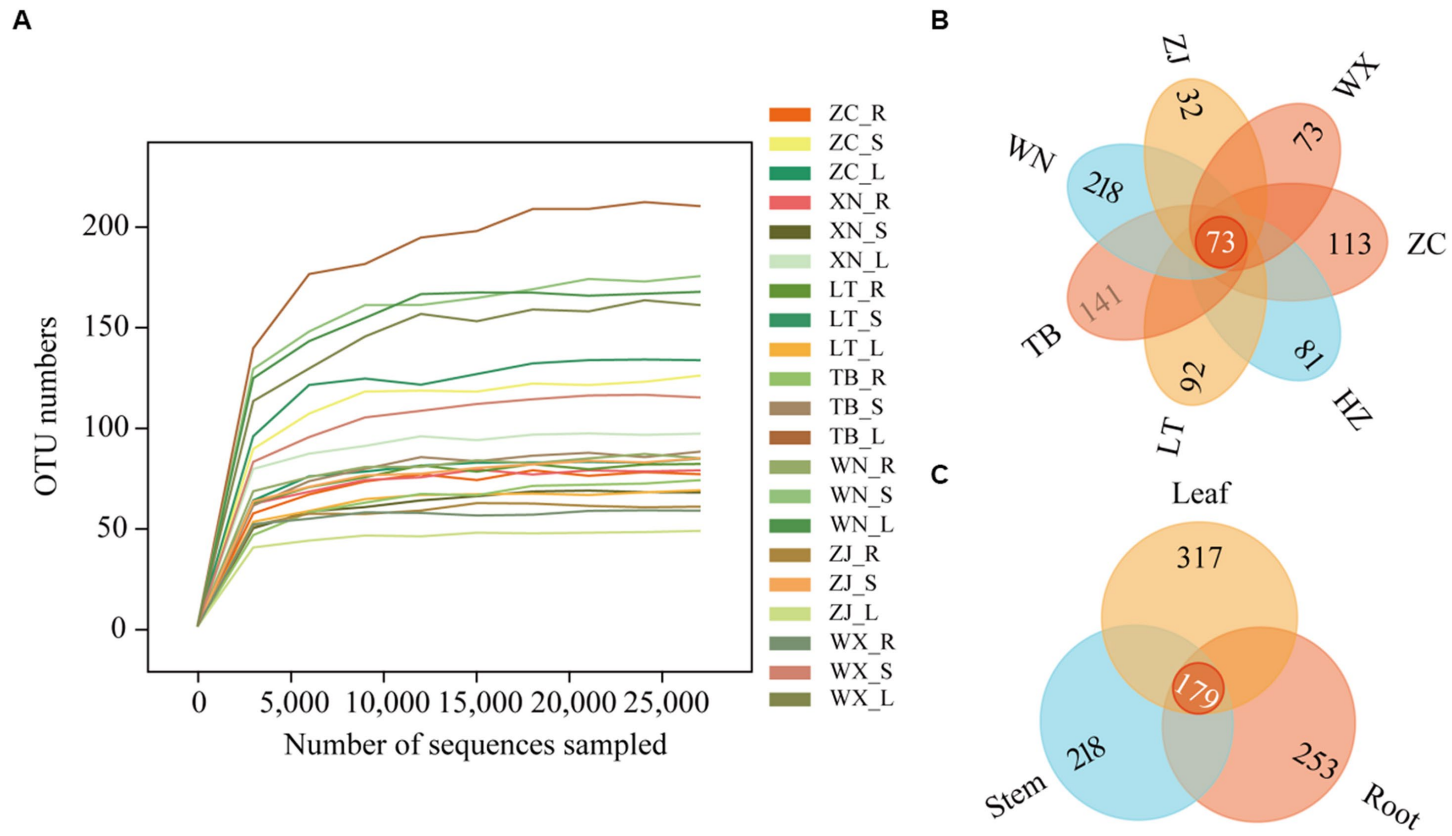
Sparse curves and Venn diagram of 21 samples. The abscissa represents the ranking of operational taxonomic units (OTUs), while the ordinate represents the relative percentage of species at the classification level **(A)**. The position of the abscissa of the extension end point of the sample curve represents the number of species in each sample. **(B)** The Venn diagram describes different and common OTUs in 7 different Tartary buckwheat materials (Zhaojue, ZJ; Wenxian, WX; Zhangchuan, ZC; Xining, XN; Lintao, LT; Taibai, TB; Weining, WN). **(C)** Different and common OTUs in roots, stems, and leaves.

Through high-throughput sequencing, 4,605,862 effective reads, 4,575,604 high-quality sequences, and 823 operational taxonomic units (OTUs) were obtained from 21 Tartary buckwheat tissue samples. The length of the sequences ranged from 146 bp to 438 bp. The smoothness of the sparse curves indicated that sequencing depth influenced the diversity observed in the samples. When the sequencing depth increased, the curve depicting the number of OTUs in the 21 samples flattened out (Figure 2A). This indicated that the sequencing results captured the diversity present in the current sample, and further increasing the sequencing depth did not reveal new OTUs. The Venn diagram of OTUs (Figure 2B) showed 73 common OTUs among the 7 different varieties of Tartary buckwheat. However, each variety had unique OTUs, with ZC, XN, LT, TB, WN, ZJ, and WX having 113, 81, 92, 141, 218, 32, and 73 unique OTUs, respectively. Additionally, there were 179 OTUs shared between the root, stem, and leaf samples, with 253 unique OTUs in the root, 218 unique OTUs in the stem, and 317 unique OTUs in the leaf (Figure 2C).

The α diversity of endophytic fungi in Tartary buckwheat was assessed in seven sampling areas using measures of community richness (Chao 1 index, Observed species), coverage (Good coverage index), diversity (Shannon index, Simpson index), and evenness (Pielou’s index). As displayed in Table 1, it was evident that the roots of all seven materials had the lowest Chao 1 index and Observed_species index, while the stems and leaves exhibited higher values. Among the different samples, WN had the highest Chao 1 index and Observed_species index, whereas ZJ had the lowest Chao 1 index. The Good’s Coverage values for all 21 samples exceeded 99.8%, indicating that the sequencing achieved a high fungal coverage rate and accurately reflected the fungal communities in the samples. There were no significant differences in evenness among the 21 samples. In terms of community diversity, the roots, stems, and leaves of WN displayed high diversity, whereas ZC showed relatively low diversity.

**Table 1:** Alpha diversity index of fungal community in Tartary buckwheat.

Sample	Mean ± SD					
	Chao1	Goods_coverage	Observed_species	Pielou_e	Shannon	Simpson
ZC_R	81.92 ± 5.47	0.99962 ± 0.00023	77.17 ± 4.14	0.35 ± 0.02	1.81 ± 1.19	0.55 ± 0.05
XN_R	79.19 ± 13.42	0.99975 ± 0.00006	74.73 ± 13.25	0.50 ± 0.03	3.11 ± 0.28	0.79 ± 0.03
LT_R	81.03 ± 7.82	0.99973 ± 0.00008	77.57 ± 8.26	0.51 ± 0.03	3.19 ± 0.10	0.80 ± 0.02
TB_R	90.42 ± 6.23	0.99959 ± 0.00011	79.13 ± 8.03	0.47 ± 0.11	2.84 ± 0.79	0.78 ± 0.09
WN_R	71.60 ± 5.19	0.99972 ± 0.00011	68.23 ± 4.99	0.55 ± 0.03	3.48 ± 0.21	0.84 ± 0.04
ZJ_R	61.36 ± 3.16	0.99961 ± 0.00042	59.57 ± 2.32	0.53 ± 0.04	3.29 ± 0.25	0.81 ± 0.04
WX_R	58.01 ± 2.64	0.99972 ± 0.00029	57.77 ± 2.40	0.51 ± 0.02	3.06 ± 0.19	0.74 ± 0.05
ZC_S	158.80 ± 5.62	0.99940 ± 0.00047	147.17 ± 15.40	0.42 ± 0.07	2.79 ± 0.42	0.69 ± 0.10
XN_S	68.29 ± 8.77	0.99975 ± 0.00006	64.87 ± 7.76	0.45 ± 0.02	2.70 ± 0.17	0.75 ± 0.04
LT_S	93.84 ± 7.55	0.99933 ± 0.00051	86.80 ± 4.75	0.44 ± 0.08	3.01 ± 0.89	0.77 ± 0.08
TB_S	98.59 ± 7.89	0.99965 ± 0.00019	92.33 ± 4.68	0.41 ± 0.12	2.58 ± 0.62	0.70 ± 0.12
WN_S	275.81 ± 34.97	0.99915 ± 0.00071	261.37 ± 22.40	0.53 ± 0.10	3.74 ± 0.87	0.86 ± 0.07
ZJ_S	74.43 ± 8.41	0.99967 ± 0.00018	69.13 ± 5.55	0.40 ± 0.11	2.52 ± 0.66	0.67 ± 0.15
WX_S	113.95 ± 11.38	0.99947 ± 0.00014	105.23 ± 10.20	0.48 ± 0.05	3.22 ± 0.37	0.77 ± 0.09
ZC_L	85.05 ± 10.48	0.99970 ± 0.00014	80.20 ± 8.92	0.44 ± 0.08	2.77 ± 0.48	0.74 ± 0.09
XN_L	129.58 ± 14.63	0.99977 ± 0.00027	127.07 ± 15.59	0.40 ± 0.08	2.59 ± 0.66	0.63 ± 0.13
LT_L	51.23 ± 2.86	0.99978 ± 0.00023	47.03 ± 5.56	0.31 ± 0.18	1.87 ± 1.14	0.49 ± 0.32
TB_L	147.66 ± 22.33	0.99899 ± 0.00075	130.20 ± 21.40	0.50 ± 0.01	3.72 ± 0.52	0.85 ± 0.03
WN_L	367.85 ± 27.59	0.99921 ± 0.00112	355.83 ± 39.03	0.47 ± 0.10	3.16 ± 0.79	0.78 ± 0.09
ZJ_L	48.87 ± 9.45	0.99987 ± 0.00009	47.03 ± 8.16	0.47 ± 0.06	2.60 ± 0.23	0.75 ± 0.05
WX_L	179.14 ± 17.10	0.99914 ± 0.00016	157.67 ± 19.89	0.45 ± 0.13	3.27 ± 1.11	0.72 ± 0.18

Zhaojue, ZJ; Wenxian, WX; Zhangchuan, ZC; Xining, XN; Lintao, LT; Taibai, TB; Weining, WN.

**Figure 3:**
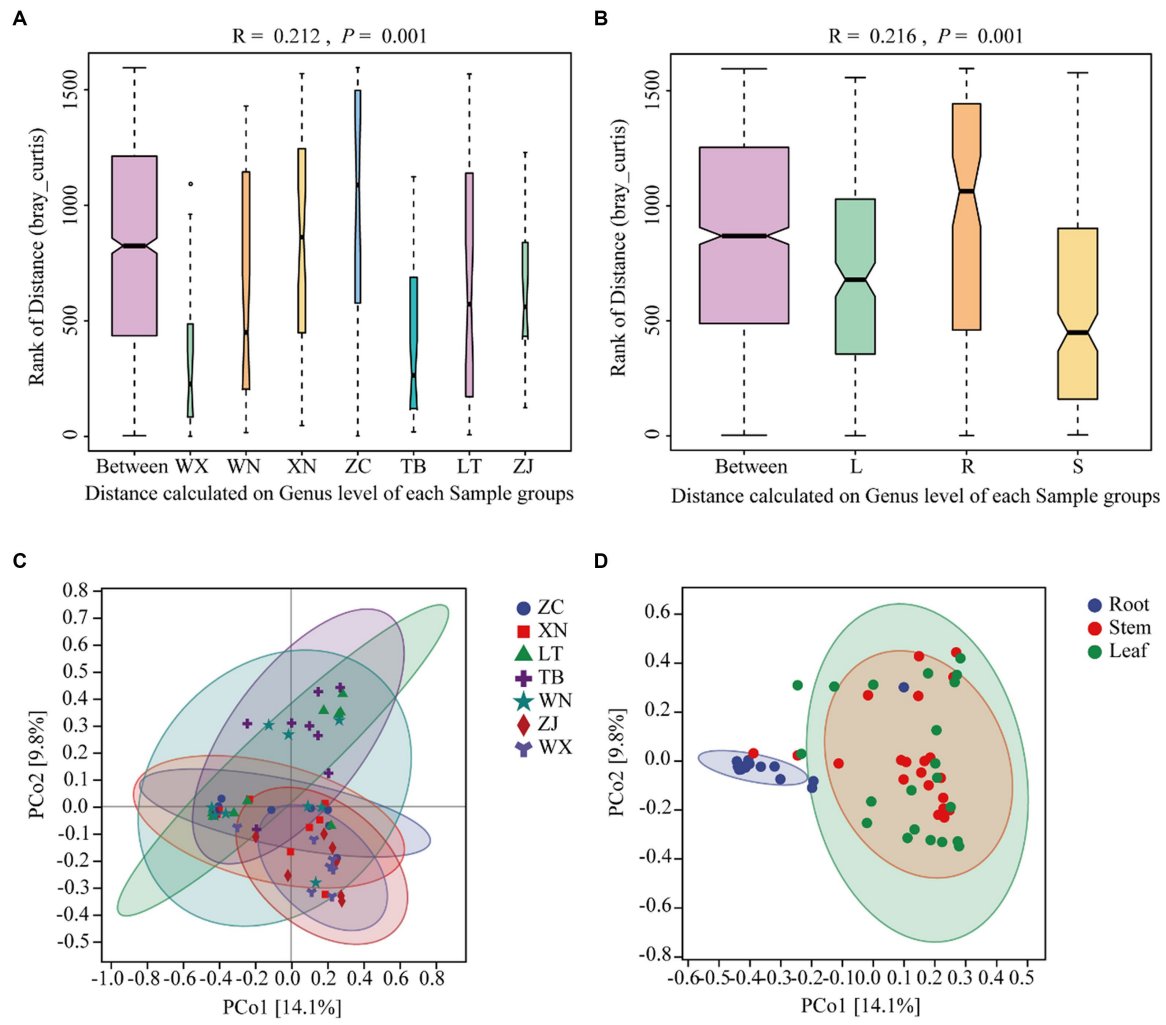
Similarity ANOSIM analysis and PCoA analysis of the endophytic fungal community in Tartary buckwheat based on Bray-Curtis distance. **(A,C)** ANOSIM analysis and PCoA analysis of 7 different Tartary buckwheat materials. **(B,D)** ANOSIM analysis and PCOA analysis of different tissue parts (roots, stems, and leaves).

Beta diversity is a measure used to assess the differences in species composition across various habitats. Higher beta diversity indicates greater dissimilarity in species composition among habitats. In this study, ANOSIM analysis was conducted using the Bray-Curtis distance algorithm to cluster the endophytic fungal communities of Tartary buckwheat in seven sample plots. The results of the ANOSIM analysis revealed that the *R*-value of Tartary buckwheat from different collection sites was 0.210 (*p* = 0.001), indicating a significant difference in species composition between the sample groups compared to within the group (Figure 3A). Similarly, the ANOSIM analysis of different tissue parts (Figure 3B) yielded an *R*-value of 0.216 (*p* = 0.001), indicating significant variation in endophytic fungal diversity among different tissue parts. PCoA analysis further demonstrated differences in the endophytic fungal communities of Tartary buckwheat among regions, with the PCo1 axis explaining 14.1% of the data and the PCo2 axis explaining 9.8% of the data (Figure 3C). Specifically, the samples from WX and ZJ displayed a relatively compact clustering pattern, suggesting similar endophytic fungal communities. ZC and XN samples also exhibited a compact grouping. However, the samples from other plots displayed a looser clustering pattern, indicating differences in endophytic fungal diversity. Similarly, there were differences observed in the distribution of endophytic fungi among different tissue parts of Tartary buckwheat (Figure 3D). The distribution of endophytic fungi in the roots appeared compact, with a relatively large distance observed between the roots and the stems or leaves, indicating significant differences in endophytic fungi between the root and the stem/leaf. Conversely, the distribution of endophytic fungi in the stems and leaves appeared looser and closer, suggesting a similar diversity of endophytic fungi within the stems and leaves.

### 3.2 Composition of endophytic fungal communities in Tartary buckwheat

**Figure 4:**
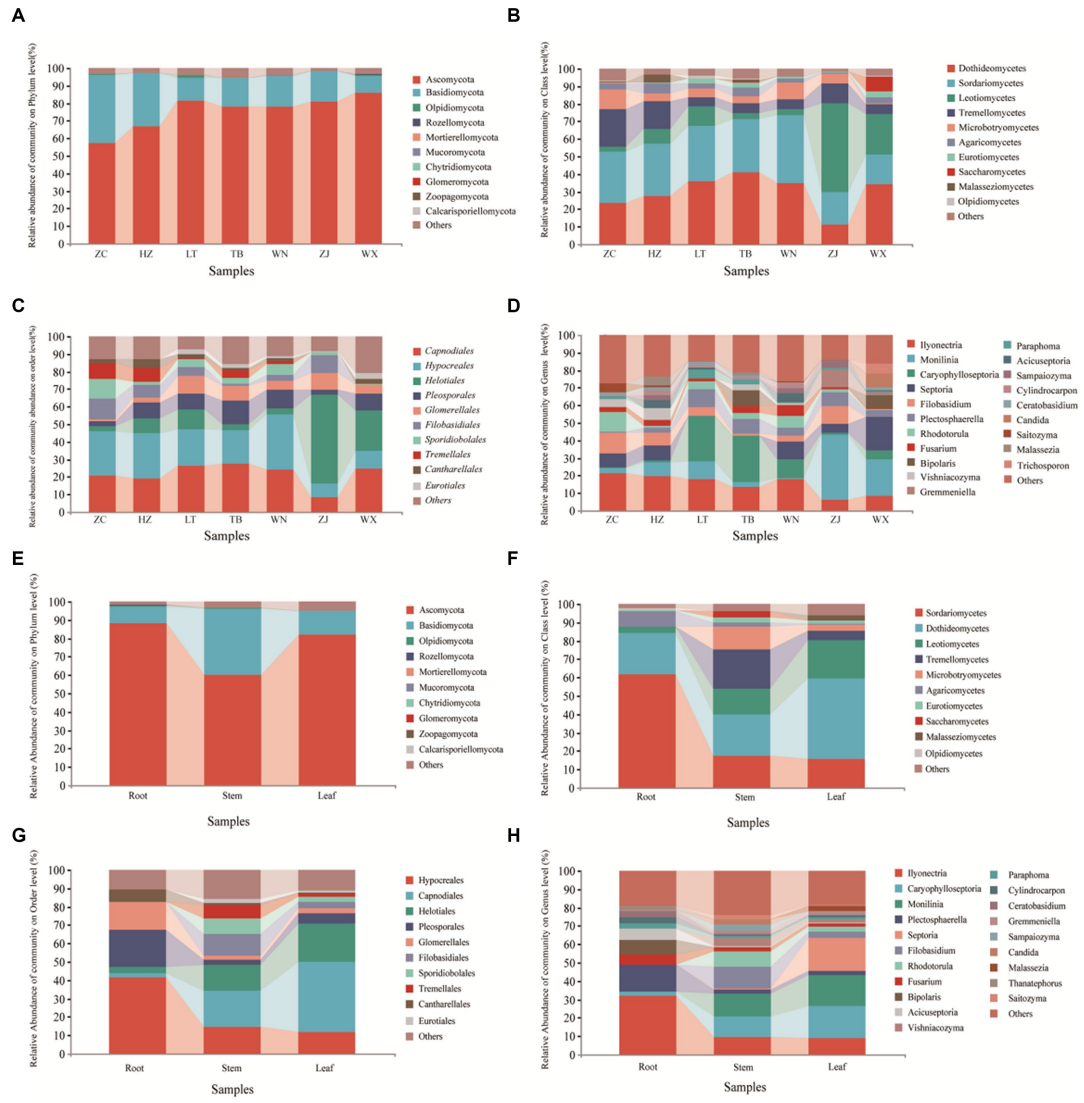
Composition of endophytic fungal community of Tartary buckwheat. The first four figures represent the composition of endophytic fungi at the phylum level **(A)**, class level **(B)**, order level **(C)**, and genus level **(D)** for different samples. The following four figures represent the composition of endophytic fungi at the phylum level **(E)**, class level **(F)**, order level **(G)**, and genus level **(H)** in different tissue parts.

The classification chart of the top 10 phyla levels of abundance revealed the dominance of *Ascomycota* as the most prevalent phylum among endophytic fungi in Tartary buckwheat, with relative abundances ranging from 57.47 to 86.21% (Figure 4A), followed by *Basidiomycota*. These two phyla encompassed the majority of the endophytic fungi, while other phyla had minimal representation. At the class level (Figure 4B), *Sortariomycetes* was the dominant class in ZC, XN, and WN, accounting for 29.26, 29.35, and 38.76% of the relative abundances, respectively. Conversely, *Dothideomycetes* was the dominant class in LT, TB, and WX, comprising 35.81, 40.97, and 34.40% of the relative abundances, respectively. In ZJ, *Leotiomycetes* was the dominant class, with a relative abundance of 50.75%. At the order level (Figure 4C), *Hypocreales* was the dominant order in ZC, XN, and WN, with relative abundances of 25.41, 25.87, and 31.46%, respectively. Meanwhile, *Capnodiales* prevailed in LT, TB, and WX, representing 26.52, 27.38, and 24.92% of the relative abundances, respectively. In ZJ, the dominant order was *Helotiales*, with a relative abundance of 50.75%. At the genus level (Figure 4D), *Ilyonectria* was the dominant genus in ZC, XN, and WN, with relative abundances of 21.38, 19.485, and 17.87%, respectively. *Caryophylloseptoria* prevailed in LT and TB, accounting for 25.86 and 26.46% of the relative abundances, respectively. In ZJ and WX, *Monilinia* dominates, representing 37.06 and 20.99% of the relative abundances, respectively.

Upon examination of different tissue parts of Tartary buckwheat, it was observed that the dominant phylum in the roots, stems, and leaves was *Ascomycota*, with relative abundances of 88.47, 60.24, and 81.92%, respectively (Figure 4E). At the class level (Figure 4F), *Sortariomycetes* prevailed in the roots, accounting for 61.60% of the relative abundance. In the stems and leaves, *Dothideomycetes* was the dominant class, representing relative abundances of 22.34 and 44.10%, respectively. When considering the order level (Figure 4G), *Hypocreales* emerged as the dominant order in the roots, with a relative abundance of 41.49%. In contrast, *Capnodiales* dominated in the stems and leaves, exhibiting relative abundances of 19.66 and 38.18%, respectively. At the genus level (Figure 4H), *Ilyonectria* prevailed in the roots, accounting for 31.89% of the relative abundance. *Monilinia* emerged as the dominant genus in the stems, constituting 12.43% of the relative abundance. For the leaves, *Caryophylloseptoria* demonstrated dominance with a relative richness of 17.44%.

### 3.3 The effect of endophytic fungi on the content of flavonoid metabolites in Tartary buckwheat

**Figure 5:**
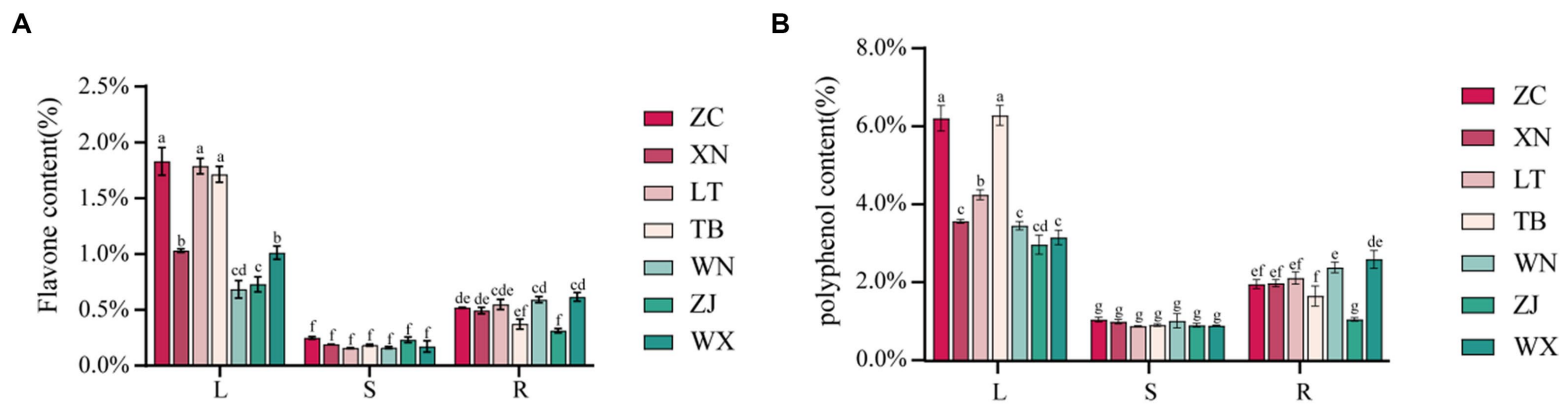
Content map of flavonoids and polyphenols in 21 Tartary buckwheat samples (Zhaojue, ZJ; Wenxian, WX; Zhangchuan, ZC; Xining, XN; Lintao, LT; Taibai, TB; Weining, WN). (A) Flavone content in 21 Tartary buckwheat samples. (B) Polyphenol content in 21 Tartary buckwheat samples.

The total flavonoid content in the roots, stems, and leaves of the seven samples was determined using the NaNO_2_-Al(NO_3_)_3_ method, as illustrated in Figure 5A. The distribution of total flavonoid content was summarized as follows: leaves>roots>stems. Among the various leaf parts, ZC, LT, and TB displayed the highest total flavonoid content, with values of 1.830, 1.787, and 1.714%, respectively, while XN and WX showed slightly lower levels of 1.030 and 1.011%, respectively. WN and ZJ exhibited the lowest content, measuring less than 1%. In the roots, ZC, XN, LT, WN, and WX displayed relatively high total flavonoid content, with values of 0.519, 0.492, 0.549, 0.591, and 0.616%, respectively, showing no significant difference. Conversely, TB and ZJ showed lower levels of total flavonoid content, measuring less than 0.4%. The stems generally exhibited low total flavonoid content, ranging from 0.156 to 0.232%, with no significant differences observed among the seven types of Tartary buckwheat stems. The total phenolic content in the roots, stems, and leaves of the seven samples was determined using the Folin phenol method. Figure 5B presents the results, illustrating that the approximate distribution of total phenolic content followed a similar pattern: leaves>roots>stems. Among the leaves, ZC and TB presented the highest total phenolic content, with values of 6.234 and 6.313%, respectively, while LT exhibited a slightly lower level of 4.279%. The total phenolic content of XN, WN, ZJ, and WX ranged from 2.997 to 3.594%, indicating the lowest values. In the roots, ZC, XN, LT, WN, and WX displayed relatively high total phenolic content, measuring 1.986, 2.009, 2.142, 2.412, and 2.620%, respectively. Conversely, TB and ZJ showed lower levels of total phenolic content at 1.683 and 1.084%, respectively. The stems demonstrated generally low levels of polyphenols, ranging from 0.909 to 1.076%. Overall, the trends in total flavonoid and total phenolic content among the different parts of the seven samples were consistent. Specifically, ZC exhibited the highest total flavonoid and phenolic content in its roots, stems, and leaves. The LT leaves displayed higher total flavonoid content, but lower total phenolic content. WN and ZJ generally showed low levels of total flavonoid and phenolic content.

**Figure 6:**
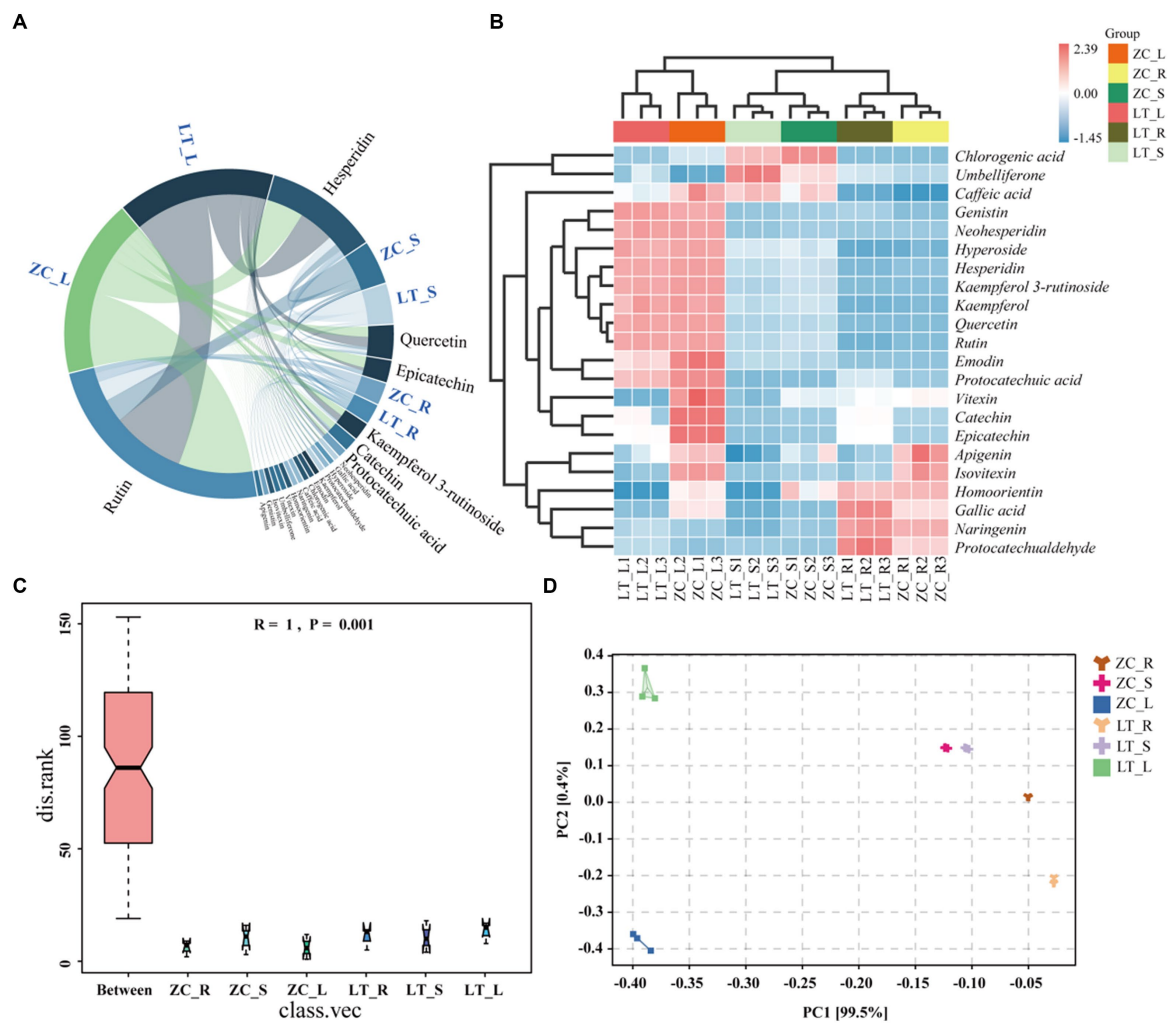
Chord diagram, heat maps, ANOSIM analyses, and PCA analyses of 22 flavonoid metabolites in ZC and LT roots, stems, and leaves. **(A)** The chord diagram shows the proportion of 22 flavonoids in the roots, stems, and leaves of ZC and TL. **(B)** The heat map intuitively reflects the differences in the content of 22 flavonoids in different samples. In the heat map, red indicates a content higher than the average, while blue indicates a content lower than the average. The darker the color, the more significant the difference. **(C,D)** ANOSIM and PCA analyses of flavonoid metabolite content in roots (R), stems (S), and leaves (L) of tissue samples from ZC and LT.

Numerous studies have demonstrated the influence of endophytic fungi on the secondary metabolite content in plants (Shen et al., 2022; Lu et al., 2023). The results of this study indicated significant differences in the composition and abundance of endophytic fungi among the seven distinct collection sites of Tartary buckwheat materials. These differences also translated into variations in the total flavonoid and phenol content within the plants. Therefore, we hypothesized that specific endophytic fungi in Tartary buckwheat contributed to the discrepancies in secondary metabolite content. To validate this hypothesis and identify the precise metabolites affected by endophytic fungi, we selected ZC and LT, which displayed the greatest discrepancy in fungi composition as well as significant differences in flavonoid and polyphenol content. Subsequently, the content of 40 flavonoids in the roots, stems, and leaves of these two materials was examined. As depicted in Figure 6A, 22 flavonoids were detected from the roots, stems, and leaves of ZC and LT (Tartary buckwheat does not contain the other 18 flavonoid metabolites, Supplementary Table S3). The levels of these flavonoids ranged from highest to lowest as follows: Rutin, Hesperidin, Quercetin, Epicatechin, Kaempferol 3-rutinoside, Catechin, Protocatechuic acid, Neohesperidin, Gallic acid, Hyperoside, Protocatechualdehyde, Kaempferol, Emodin, Chlorogenic acid, Caffeic acid, Naringenin, Homoorientin, Vitexin, Umbelliferone, Isovitexin, Genistin, and Apigenin. The heat map demonstrated significant variations in metabolite content across the roots, stems, and leaves of Tartary buckwheat (Figure 6B). For instance, the content of Genistin, Neohesperidin, Hyperoside, Hesperidin, Kaempferol 3-rutinoside, Kaempferol, Quercetin, Rutin, Emodin, and Protocatechuic acid in the leaves was notably higher than in the stems and roots. Furthermore, Chlorogenic acid, Umbelliferone, and Caffeic acid exhibited the highest content in the stems, while Naringenin and Protocatechualdehyde was significantly higher in the roots compared to the leaves and stems. Notably, there were distinctive differences in the content of flavonoid metabolites among different Tartary buckwheat materials. For example, ZC leaves displayed higher levels of Caffeic acid, Vitexin, Catechin, Epicatechin, Apigenin, Isovitexin, Homoorientin, and Gallic acid compared to LT. Additionally, ZC roots also contained greater amounts of Apigenin and Isovitexin. The results of the ANOSIM analysis revealed that the *R*-value of different tissue samples of ZC and LT was 1 (*p* = 0.001), indicating that the differences in flavonoid metabolite content between tissue samples were highly significantly greater than within samples (Figure 6C). PCA analysis visualized this intergroup difference, with the PC1 axis explaining 99.5% of the data and the PC2 explaining 0.4% of the data (Figure 6D). It is easy to see in the figure that flavonoid metabolite content is more similar between different samples of the same tissue, such as within roots, stems, and leaves. On the contrary, the content of flavonoid metabolites varied very much between different tissue samples of the same sample, especially in leaves compared to roots and stems.

**Figure 7:**
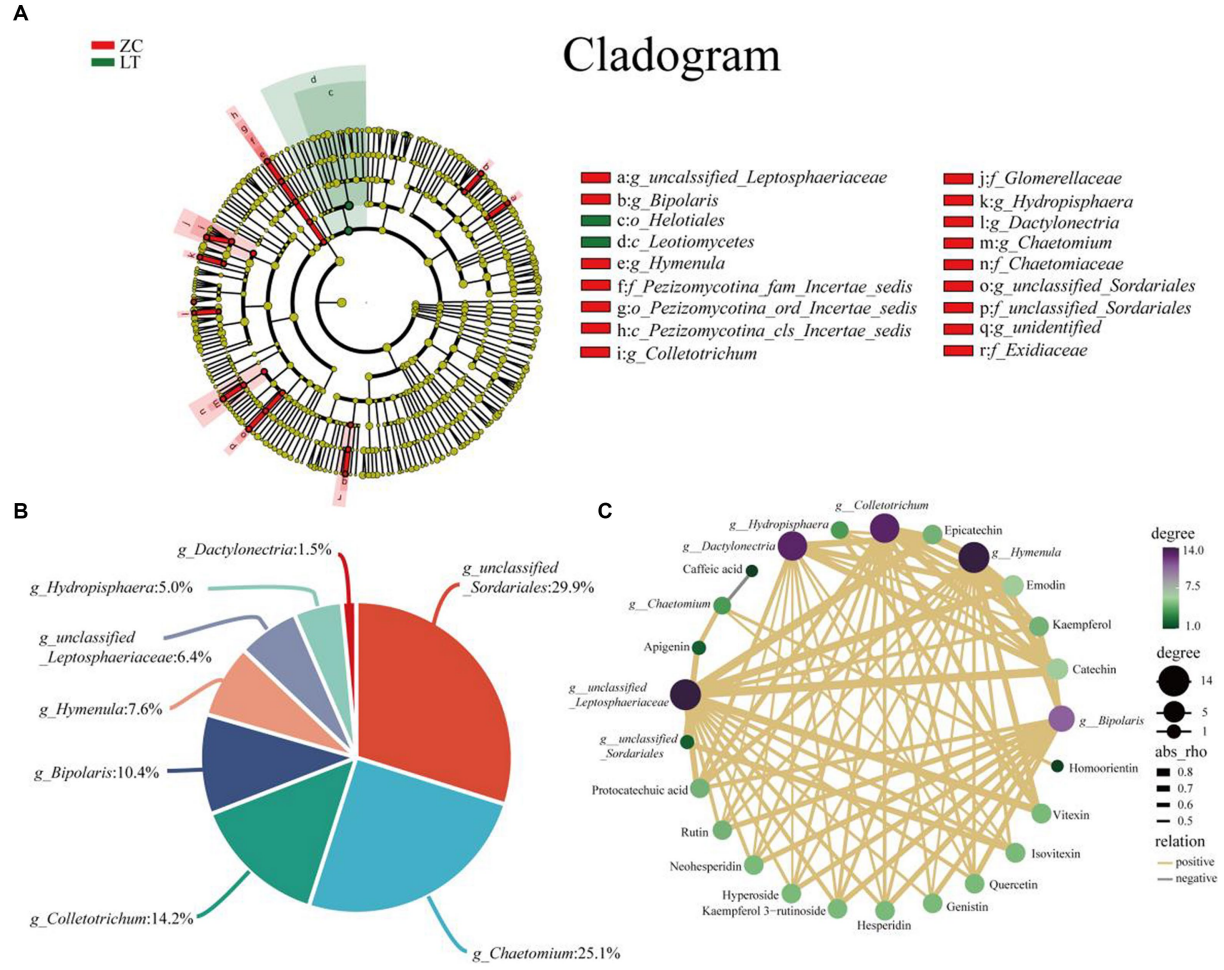
Analysis of differences in endophytic fungi between ZC and LT, as well as correlation between flavonoids and different endophytic fungi at the genus level. **(A)** LEfSe analysis shows the hierarchical relationships of the main taxonomic units in the sample community from phylum to genus (from inner circle to outer circle). The node size corresponds to the average relative abundance of the classification unit; A node with a red background indicates a higher abundance of the classification unit in ZC, while a node with a green background indicates a higher abundance of the classification unit in LT. The letters indicate the names of taxonomic units with significant differences between groups. **(B)** Percentage of endophytic fungi with genus level differences. **(C)** Association network diagram of endophytic fungi and flavonoid metabolites with significant correlation.

To examine the variations in endophytic fungi between ZC and LT, we performed LEfSe analysis on their respective populations. LEfSe analysis is a differential analysis method that enables simultaneous analysis across all classification levels. Additionally, it emphasizes the identification of robust differential species, referred to as biomarkers. The results of the LEfSe analysis (Figure 7A) revealed significant differences in the endophytic fungi of ZC and LT at the class, order, family, and genus levels. Notably, there were nine significantly different genus-level classifications, including *unclassified_Liptosphaeriaceae*, *Bipolaris*, *Hymenula*, *Colletotrichum*, *Hydroptosphaera*, *Dactylonectria*, *Chaetomium*, *unclassified_Sortariales*, and *one unidentified taxonomic unit* (g_identified). Among these nine distinct genus-level taxonomic units, *unclassified_Sordariales* accounted for the largest proportion at 29.9%, followed by *Chaetomium* (25.1%), *Colletotrichum* (14.2%), *Bipolaris* (10.4%), *Hymenula* (7.6%), *unclassified_Lettosphaeriaceae* (6.4%), *Hydroptosphaera* (5.0%), and *Dactylonectria* (1.5%) (Figure 7B). Furthermore, Pearson correlation analysis was conducted to examine the relationship between endophytic fungi and flavonoid metabolite content at the genus level. Supplementary Figure S2 displays the complete correlation heatmap, while Figure 7C presents a network diagram highlighting the significant correlations between fungi and flavonoid metabolites. The correlation network diagram distinctly demonstrated the impact of different genus-level endophytic fungi on flavonoid content. *Bipolaris*, *Hymenula*, *Colletotrichum*, *Dactylonectria*, and *unclassified_Lettosphaeriaceae* exhibited significant positive correlations with the content of 11 or more flavonoids, including Rutin, Hesperidin, Quercetin, Epicatechin, Kaempferol 3-rutinoside, Catechin, Protocatechuic acid, and others. *Hydroptosphaera* and *Chaetomium* displayed significant positive correlations with the content of four and three flavonoid metabolites, respectively. Additionally, *Chaetomium* exhibited a significant negative correlation with the content of Caffeic acid. *Unclassified_Sordariales* demonstrated a significant positive correlation with the content of two flavonoid metabolites.

### 3.4 The effect of endophytic fungi on the phenotypic characters of Tartary buckwheat

Various biological characteristics of 7 plant samples were measured, namely plant height, middle branch length, number of main stem nodes, number of main stem branches, stem thickness, branch angle, leaf length, and leaf width (Table 2). Plant height serves as an intuitive indicator of plant growth and speed (Zhao et al., 2022). Notably, significant differences in plant height were observed among the seven collection locations of Tartary buckwheat. Specifically, TB and WX exhibited higher plant heights at 145.33 cm and 109.33 cm, respectively. Furthermore, TB demonstrated significantly taller plants compared to the other six samples. Conversely, ZJ plants exhibited the shortest height at only 54.33 cm. The length of the middle branch, number of main stem nodes, number of main stem branches, and stem thickness are indicative of plant characteristics along the stem. Notably, WX had the longest middle branch, measuring 83.33 cm, which was significantly longer than the other six species. The remaining Tartary buckwheat samples displayed middle branch lengths ranging from 5.33 cm to 39.17 cm. Additionally, TB boasted the highest number of main stem nodes, averaging 18 nodes per plant. The distribution of node numbers among the other plants was relatively uniform, ranging from 10.33 to 14.66. As for the number of branches on the main stem, LT exhibited the highest count, with an average of 10.33 branches per plant. In contrast, ZJ and WX had the lowest branch counts, averaging only 4 branches per plant. Similarly, LT showcased the greatest stem thickness at 7.33 mm, while WX displayed a stem thickness of only 4.33 mm. In terms of branching angles, XN and WX boasted the highest angles, measuring 55.00° and 44.33°, respectively. ZJ, LT, WN, and TB followed with branching angles of 30.33°, 28.33°, 27.67°, and 21.67°, respectively. Remarkably, ZC exhibited the smallest branch angle, measuring only 18.00°. Regarding leaf characteristics, TB possessed the largest leaves, with lengths and widths measuring 6.00 cm and 7.13 cm, respectively. WX and XN closely followed, followed by ZJ, LT, and WN, with ZC exhibiting the smallest leaf size.

**Table 2:** Statistical table of phenotypic characters of 7 Tartary buckwheat samples.

Sample	Mean ± SD(n = 3)							
	Plant height (cm)	Length of middle branches (cm)	Number of main stem nodes	Number of branches on the main stem	Stem thickness (mm)	Branching angle	Leaf length (cm)	Leaf width (cm)
ZC	87.00 ± 8.89 c	17.67 ± 5.03 cd	13.00 ± 1.73 bc	7.67 ± 1.15 b	6.67 ± 1.15 ab	18.00 ± 3.00 c	2.87 ± 0.35 d	2.90 ± 0.36 c
XN	85.67 ± 6.66 c	5.33 ± 1.53 d	12.33 ± 2.08 bc	8.00 ± 1.73 b	5.33 ± 1.53 bc	55.00 ± 5.00 a	5.33 ± 1.24 ab	4.70 ± 1.21 b
LT	96.00 ± 3.00 bc	16.00 ± 6.56 d	14.67 ± 1.15 b	10.33 ± 1.15 a	7.33 ± 0.58 a	28.33 ± 7.02 c	3.70 ± 0.26 cd	4.17 ± 0.29 bc
TB	145.33 ± 10.02 a	11.67 ± 0.58 d	18.00 ± 1.00 a	4.67 ± 0.58 cd	6.67 ± 1.15 ab	21.67 ± 2.89 c	6.00 ± 0.40 a	7.13 ± 0.90 a
WN	90.00 ± 2.65 c	39.17 ± 9.46 b	14.00 ± 1.00 b	6.33 ± 1.15 bc	5.33 ± 0.58 bc	27.67 ± 6.81 c	4.27 ± 0.42 bc	4.93 ± 1.08 b
ZJ	54.33 ± 2.08 d	32.00 ± 1.00 bc	10.33 ± 2.89 c	4.00 ± 1.00 d	5.33 ± 1.15 bc	40.33 ± 0.58 b	3.10 ± 0.17 cd	3.73 ± 0.40 bc
WX	109.33 ± 13.80 b	83.33 ± 17.95 a	14.33 ± 2.31 b	4.67 ± 0.58 cd	4.33 ± 0.58 c	44.33 ± 9.29 b	5.27 ± 1.12 ab	4.83 ± 0.29 b

Zhaojue, ZJ; Wenxian, WX; Zhangchuan, ZC; Xining, XN; Lintao, LT; Taibai, TB; Weining, WN. The different letters (a, b, c, and d) indicate significance at *p* = 0.05 level.

**Figure 8:**
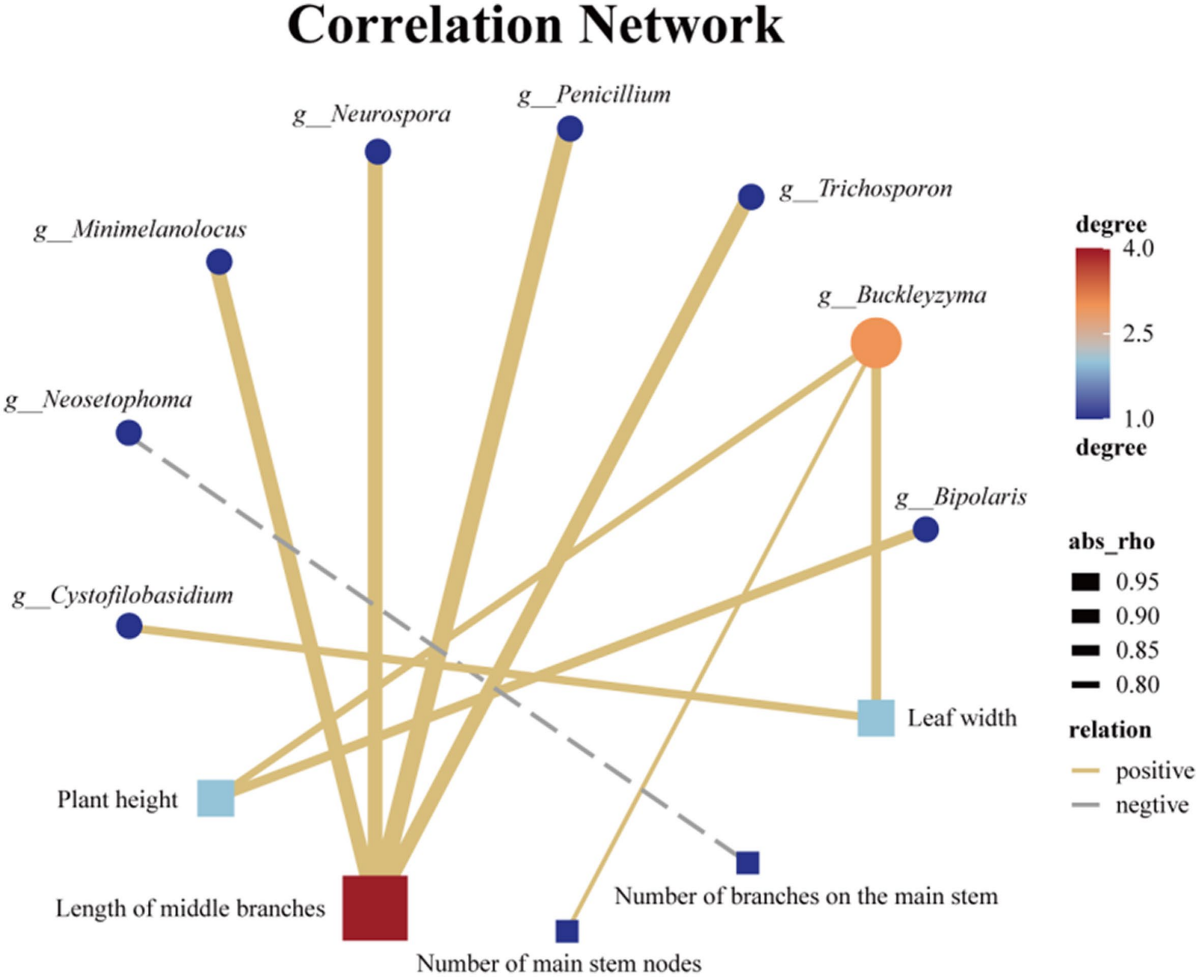
Association network diagram of Endophytic fungi with differences at the genus level and phenotypic traits with significant correlation.

Endophytic fungi play a crucial role throughout the entire life cycle of plants, influencing their growth, development, and metabolic processes (Kandar et al., 2018; Yan et al., 2019; Wang et al., 2022). Therefore, it is highly plausible that the endophytic fungi found in Tartary buckwheat have an impact on various phenotypic traits, including plant height, stem diameter, and number of branches. Similarly, LEfse analysis was employed to identify endophytic fungal species exhibiting significant differences among the 7 samples. The distinct distribution of endophytic fungi across the 7 samples was observed at various taxonomic levels, including 1 phylum, 3 classes, 7 orders, 13 families, and 24 genera, with the inclusion of several unidentified endophytic fungal taxonomic units (Supplementary Figure S3). Moreover, a correlation analysis was conducted between the 21 different genus-level endophytic fungi and 8 biological traits of Tartary buckwheat (Supplementary Figure S4). Initially, we discovered a significant positive correlation between plant height and *Buckleyzyma* and *Bipolaris* (Figure 8). Furthermore, the length of the middle branch exhibited a significant positive correlation with multiple genera of endophytic fungi, such as *Penicillium*, *Minimelanolocus*, *Neurospora*, *Trichosporon*, and one unidentified endophytic fungal taxon. The number of main stem branches showed a significant negative correlation with *Neosetophoma* while displaying a significant positive correlation with an unidentified endophytic fungal taxon. Leaf width displayed a significant positive correlation with *Buckleyzyma* and *Cystofilobasidium*. Notably, no significant correlation was observed between endophytic fungi and stem diameter, number of main stem nodes, leaf length, and branching angle at the genus level.

## 4 Discussion

Extensive research on fungal plant interactions has focused on endophytic fungi due to their ability to increase the content of secondary metabolites, enhance biocontrol capabilities, and promote host growth and development (Kumar and Verma, 2018; Mishra et al., 2021; Zhang et al., 2022). For instance, *Alternaria* in *S. alopecuroides* seeds has been identified as a potential promoter for the accumulation of bioactive compounds in the host (Ju et al., 2022). Wild halophytic rice *Oryza coarctata* showed significant enhancement in root length, stem length, and total tiller number after inoculation with the endophytic fungus *Aspergillus welwitschiae* Ocstreb1 (AwOcstreb1) (Airin et al., 2023). In other crop plants such as maize, bean, and soybean, plant height and dry weight were also significantly increased after inoculation with the fungi *Purpureocillium* spp. and *M. marquandii strains* (Baron et al., 2020). Furthermore, inoculation with specific endophytic fungi, such as *Alternaria chlamydospora*, *Piriformospora indica*, and *Trichoderma longibrachiatum*, has been shown to impact the plant height, stem diameter, number of branches, and root length of the host (Kandar et al., 2018; Abdolmaleki et al., 2022; Wang et al., 2022; Zuo et al., 2022). In the current study, a comprehensive correlation analysis was conducted between endophytic fungi in Tartary buckwheat and both flavonoid metabolite content and phenotypic traits. Notably, we observed a strong positive correlation between the *Bipolaris* genus and the content of 11 flavonoid metabolites, including key metabolites like rutin and quercetin. Additionally, a significant positive correlation with plant height was uncovered. These findings suggest that endophytic fungi belonging to the *Bipolaris* genus play a crucial role in the modulation of Tartary buckwheat. Consequently, this provides new avenues for exploring functional strains that produce flavonoids from *Fagopyrum tataricum*, utilizing wild resources, and implementing biological fertilizer strategies to enhance its quality and yield.

High-throughput sequencing technology has found extensive utilization in microbial sequencing, enabling the comprehension of microorganism species composition and abundance across different plants, thereby shedding light on microbial diversity and its dynamic patterns. Additionally, it provides insights into the functional potential of microorganisms within the communities, aiding the understanding of their roles and interrelationships within ecosystems (Baldrian et al., 2022). In this study, MiSeq high-throughput technology was employed to sequence the ITS rDNA genes of endophytic fungi in 21 samples of Tartary buckwheat from 7 distribution plots in central and southern China. This approach comprehensively captured the community structure and diversity of endophytic fungi present in Tartary buckwheat. The findings revealed that WN exhibited the highest richness of endophytic fungal communities in both the stems and leaves, significantly surpassing other groups. Additionally, WN demonstrated high uniformity and diversity. Conversely, ZJ generally displayed lower community richness, as well as lower evenness and diversity within its stems and leaves. No significant differences in diversity indices were observed among the other samples, potentially attributable to variations in climate, soil composition, and surrounding plant diversity across different geographical locations. Research conducted by Wang et al. (2023) highlighted the importance of temperature thresholds in shaping changes in root-associated endophytic fungal diversity. Beyond these thresholds, the abundance of OTUs colonizing root tissue rapidly decreases. Remarkably, habitats characterized by high soil pH and low nutrient availability tend to exhibit greater fungal diversity. Moreover, the species composition and quantity of accompanying plants also impact the diversity and richness of endophytic fungi within the host plant (Yokoya et al., 2017; Fu et al., 2022).

The distribution of endophytic fungi is tissue-specific, as observed in *Azadirachta Indica* where the richness and diversity of endophytic fungi vary across the roots, leaves, stems, bark, and branches (Chutulo and Chalannavar, 2018). Hence, this study aimed to comprehensively assess the diversity and richness of endophytic fungi in Tartary buckwheat by selecting seven different sampling locations and three distinct tissues. Regardless of the sampling location or tissue, the predominant classification at the phylum level was *Ascomycota*, which is the largest classification level among fungi and encompasses approximately 64,000 known species (Bennett and Turgeon, 2016). The dominant genera of endophytic fungi found in various sampling sites and tissues included *Ilyonectria*, *Monilinia*, and *Caryophylloseptoria*. *Ilyonectria* is associated with root rot symptoms in diverse herbaceous and woody host plants such as ginseng, Panax notoginseng, and apple (Zhu et al., 2019; Popp et al., 2020; Bischoff Nunes and Goodwin, 2022). Interestingly, it can also exist as an endophytic fungus in seemingly healthy plant roots, where it can inhibit other fungal root pathogens and maintain the health of the host (Yang et al., 2019). Based on this, we speculated that *Ilyonectria* functioned similarly in Tartary buckwheat, as no significant lesions were observed in the roots. *Monilinia* primarily inflicts damage on orchards, causing fruit brown rot (Pereira et al., 2019; Akhoon et al., 2023), while *Caryophylloseptoria* mainly leads to leaf spot disease (Yang et al., 2022). However, despite being dominant in Tartary buckwheat, these genera are hypothesized to share a similar mechanism with *Ilyonectria*. Takahiro Noda comprehensively reviewed the differences in flavonoid metabolites, such as rutin, vitexin, isovitexin, and quercetin, in various parts of Tartary buckwheat, including the roots, stems, leaves, flowers, and seeds (Takahiro et al., 2023). Notably, rutin constitutes 90% of the total phenolic content, with the highest concentration in the leaves. This finding aligns with the results of our research. The correlation analysis between flavonoids and differential fungi is presented in Figure 7.

*Colletotrichum* exhibits a significant positive correlation with the content of 13 flavonoid metabolites, suggesting its close association with flavonoid metabolism in Tartary buckwheat and its potential involvement in crucial steps of various flavonoid metabolic pathways. Fungal metabolites are typically similar to those of their hosts, indicating a close relationship between host and fungal metabolites (Johny et al., 2021). Likewise, Kim and Shim (2019) identified 25 phenolic compounds, including vitexin and skullcapflavone II, along with various terpenoids, pyranones, and other metabolites in the metabolome of *Colletotrichum*. Furthermore, two *Colletotrichum* strains isolated from *Andrographis paniculata* exhibited substantial antioxidant activity and produced high yields of total phenols and flavonoids (Li Q. et al., 2022). Another study found that after exogenous inoculation with a *Colletotrichum* strain, the content of total flavonoids, soluble total phenols, coumarins, and kaempferol increased in the body of *Annona muricata* (Rubio-Melgarejo et al., 2020). Based on the aforementioned findings, we speculated that *Colletotrichum*, as an endophytic fungus in Tartary buckwheat, was capable of producing abundant levels of flavonoids and other polyphenols. On the other hand, *Colletotrichum* can stimulate Tartary buckwheat to increase its secretion of flavonoids. Although the first aspect is easily understood, the underlying reason why *Colletotrichum* induces the release of flavonoids from Tartary buckwheat remains unclear. However, a significant amount of research has focused on *Colletotrichum*’s ability to cause anthracnose in plants, thereby posing a threat to their survival (Camiletti et al., 2022; de Aguiar Carraro et al., 2022; Willie Anderson et al., 2022). When plants encounter abiotic or biotic stress, they tend to accumulate substantial amounts of phenolic compounds in their tissues (Pedras et al., 2011). Previous studies have shown that flavonoids and other polyphenols provide some protection against certain pathogenic bacteria in the genus *Colletotrichum* (Roy et al., 2018; Tugizimana et al., 2019). For instance, during the initial stages of *Colletotrichum* infection, as mentioned earlier in *Phaseolus vulgaris* and *Annona muricata*, the levels of phenolic compounds in their tissues are upregulated. Upon infection with *Colletotrichum* in olive plants, an increase in PAL enzyme activity and *PAL* gene expression occurs, indicating that these genes play a role in olive’s defense response against anthracnose (Gouvinhas et al., 2019). PAL enzyme, the key enzyme in flavonoid synthesis and the initial enzyme in the phenylalanine pathway, catalyzes the conversion of phenylalanine to aminophenylpyruvate and ammonia (Liu et al., 2021). A second plausible speculation is that although *Colletotrichum* acts as an endophytic fungus of Tartary buckwheat rather than as an exogenous pathogen, as a parasitic fungus within the genus *Colletotrichum*, it is equally likely to trigger a certain level of chemical defense response in the plant, causing the host to secrete more phenolic compounds, a response that already exists as a norm.

Additionally, we also identified a notable positive correlation between *Bipolaris* and the content of 11 flavonoids, suggesting a potential relationship between the presence of *Bipolaris* and flavonoid levels. *Bipolaris*, specifically *Bipolaris oryzae*, is renowned for causing rice blast (Dorneles et al., 2020) as well as spot disease in diverse crops, including wheat (Zaccaron and Bluhm, 2017; Meng et al., 2020; Manzar et al., 2022). It is noteworthy that *Bipolaris* secondary metabolites are rich in flavonoids and other polyphenols, although their applications in this area still necessitate further development (Das et al., 2017; Muthukrishnan et al., 2022). Additionally, *Bipolaris* infection in wheat triggers its own SA signal transduction and WRKY33 transcription factor, enhancing the expression of phenylpropanoid pathway genes, which potentially leads to the accumulation of phenolic defense metabolites (Sahu et al., 2016). Wanke et al. (2023) have discovered that plants deposit phenolic substances at sites invaded by fungi to reinforce their cell walls and impede further fungal development. Consequently, it is plausible that *Bipolaris* may induce the host plant to secrete higher levels of polyphenols following invasion. The aforementioned studies partially elucidate the significant positive correlation between *Bipolaris* and the various flavonoid content observed in Tartary buckwheat.

Plant phenotypic traits play a vital role in the collection of wild resources (Melouk et al., 2023). Environmental factors such as altitude (Bonanomi et al., 2016), precipitation (Sun et al., 2016), and temperature (Zahedi and Sarikhani, 2016) significantly influence most morphological traits. This study focused on seven wild Tartary buckwheat samples from various habitats within the wild buckwheat germplasm resource nursery. To assess the differences, eight relevant phenotypic traits, including plant height, stem diameter, and number of main stem nodes, were measured. The aim was to examine the impact of endophytic fungi, which are consistently associated with the plant in its physiological context, by eliminating the influence of environmental factors from their native habitats. The correlation between the differential endophytic fungi and phenotypic traits in these seven Tartary buckwheat samples is shown in Figure 8. Notably, there was a significant positive correlation between *Bipolaris* and the height of Tartary buckwheat plants. Previous analyses have shown that *Bipolaris* is also significantly and positively correlated with 11 flavonoids. Under salt stress, inoculation with *Bipolaris* sp. significantly increases soybean stem length, root length, root fresh weight, and chlorophyll content (Lubna et al., 2022). The culture filtrate of *Bipolaris* is rich in indoleacetic acid, demonstrating strong free radical scavenging ability and anti-lipid peroxidation activity (Khan et al., 2017). Thus, we speculate that the endophytic fungi of the *Bipolaris* genus in Tartary buckwheat may promote its growth by secreting indoleacetic acid. Additionally, other endophytic fungi affect the plant phenotype of Tartary buckwheat, such as *Buckleyzyma*, which affects plant height and leaf width, and *Minimelanolocus*, *Neurospora*, and *Trichosporon*, which impact the length of middle branches. Limited research has been conducted on these aforementioned endophytic fungi, and currently, there are no studies discussing their impact on plant phenotypes. However, this study provides new insights into the multifaceted exploration and utilization of these endophytic fungi.

## 5 Conclusion

This study demonstrated significant differences in the plant phenotype, endophytic fungal community diversity, and flavonoid metabolite content of Tartary buckwheat from different regions. Pearson correlation analysis revealed that several endophytic fungi, including *Bipolaris*, *Hymenula*, *Colletotrichum*, *Dactylonectria*, and *unclassified_ Liptosphaeriaceae*, had the potential to enhance the content of more than 10 flavonoids, such as Rutin, Hesperidin, Quercetin, Epicatechin, Kaempferol 3-rutinoside, Catechin, and others. Moreover, *Bipolaris*, *Buckleyzyma*, *Trichosporon*, *Penicillium*, *Neurospora*, *Minimelanolocus*, and *Cystofilobasidium* exhibited the potential to promote Tartary buckwheat growth, including plant height, length of middle branches, number of main stem nodes, number of branches on the main stem, and leaf width. Particularly, *Bipolaris* not only enhanced the content of flavonoids but also stimulated growth in Tartary buckwheat. Further validation of *Bipolaris*’ functionality and utilization as a key candidate fungus for the development of biological fertilizers for Tartary buckwheat should be pursued in future research.

## Data availability statement

The datasets presented in this study can be found in online repositories. The names of the repository/repositories and accession number(s) can be found at: https://www.ncbi.nlm.nih.gov/, PRJNA1043747.

## Author contributions

MC: Conceptualization, Writing – original draft. ZD: Writing – original draft, Formal analysis. MZ: Investigation, Writing – review & editing. YS: Methodology, Writing – review & editing, Conceptualization. CL: Visualization, Writing – review & editing, Software. QL: Visualization, Writing – review & editing, Data curation. TB: Validation, Writing – review & editing, Formal analysis. ZT: Validation, Writing – review & editing. HC: Resources, Supervision, Writing – original draft.
