# Supplementary material for: The diversity of endophytic fungi in Tartary buckwheat (Fagopyrum tataricum) and its correlation with flavonoids and phenotypic traits
Source: Front Microbiol. 2024 Mar 14;15:1360988. doi: 10.3389/fmicb.2024.1360988 (PMC10979544; doi:10.3389/fmicb.2024.1360988)
Supplement: Supplementary file 1 [file Table_1.DOCX]

Supplementary Figure 1. Standard curve of 40 kinds of flavonoid standard substances.


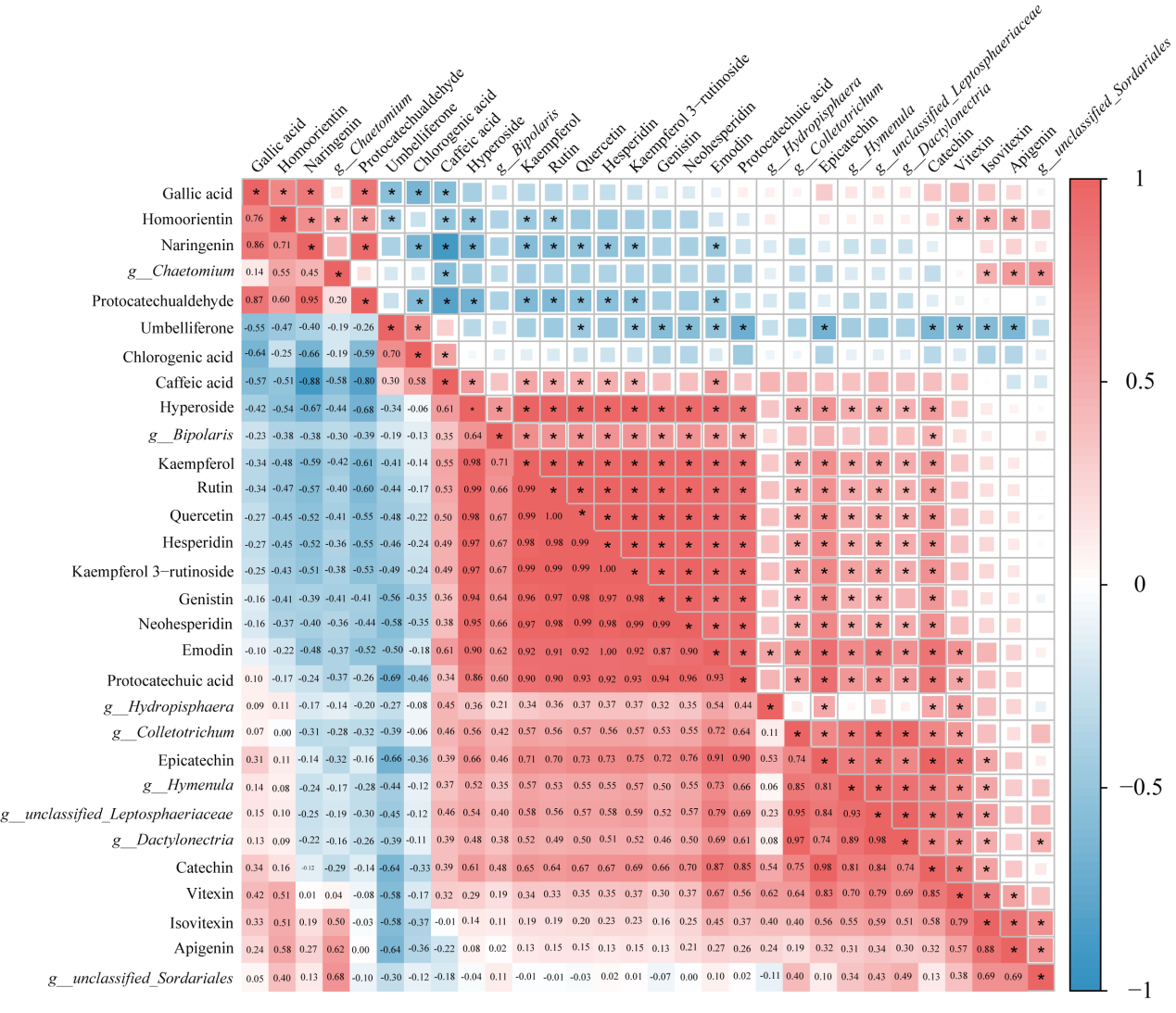


Supplementary Figure 2. Correlation analysis was conducted between eight differentially distributed genera-level endophytic fungi and the content of 22 flavonoid compounds in Tartary buckwheat.

In the above figure, red represents positive correlation, blue represents negative correlation, and the intensity of the color indicates the strength of the correlation. An asterisk (*) indicates that the correlation is statistically significant.


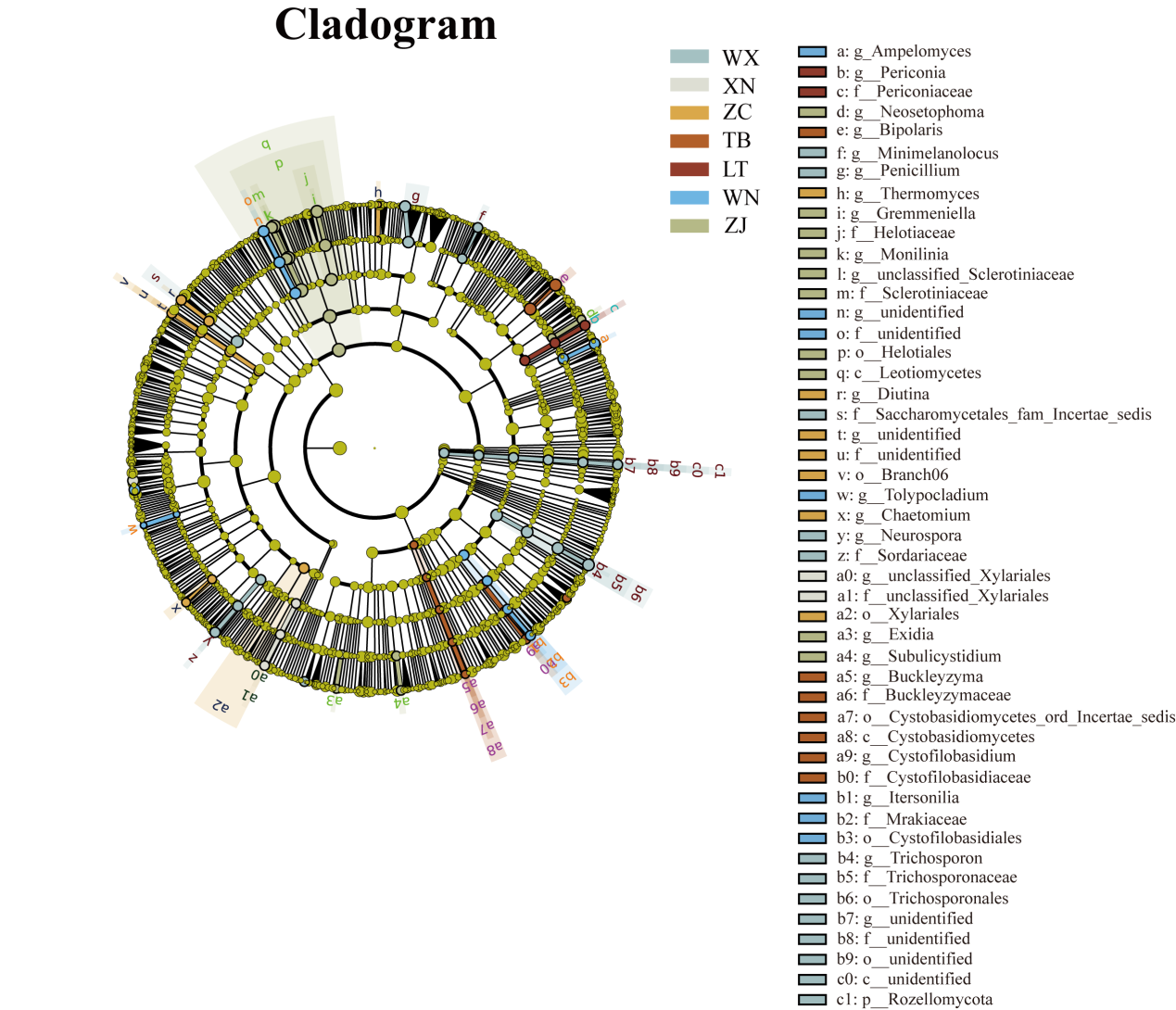


Supplementary Figure 3 Analysis of differential endophytic fungi among seven Tartary buckwheat samples.

LEfSe analysis shows the hierarchical relationships of the main taxonomic units in the sample community from phylum to genus (from inner circle to outer circle). The node size corresponds to the average relative abundance of the classification unit. Different colors represent 7 different Tartary buckwheat samples. The letters indicate the names of taxonomic units with significant differences between groups.


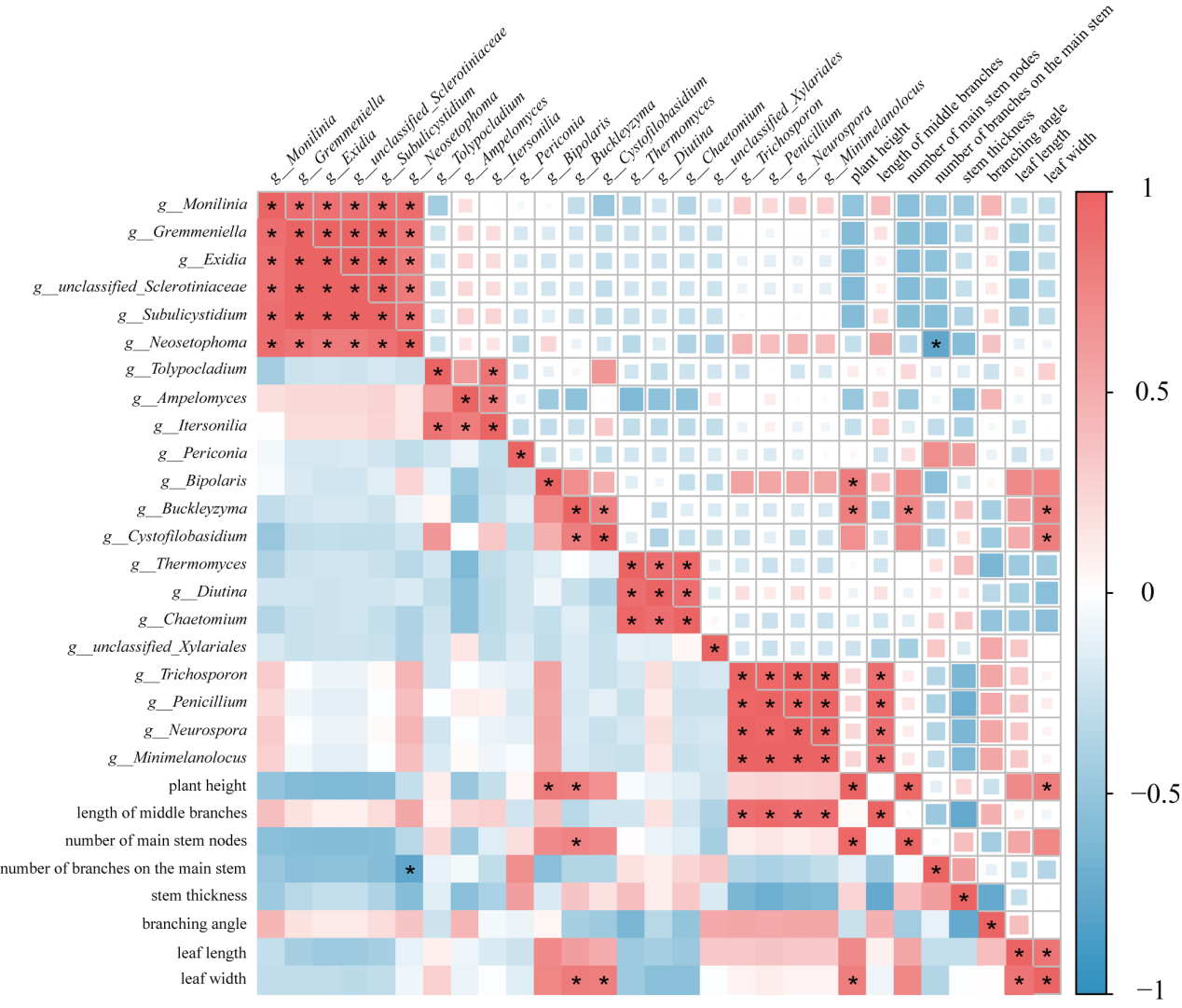


Supplementary Figure 4 Correlation analysis between 21 differentially distributed genera-level endophytic fungi and the eight phenotypic traits of Tartary buckwheat samples..

In the above figure, red represents positive correlation, blue represents negative correlation, and the intensity of the color indicates the strength of the correlation. An asterisk (*) indicates that the correlation is statistically significant.

Note: A total of 24 genus-level differential endophytic fungi were analyzed in Supplementary Figure 3, but three of these did not identify species (n:g _unidentified, t:g _unidentified, b7:g _unidentified), so only 21 differential genus-level endophytic fungi were utilized in the correlation analysis.
